# *ISupport-Brasil*: Preliminary results of the usability and acceptability assessment by caregivers of people who have dementia

**DOI:** 10.3389/fmed.2022.981748

**Published:** 2022-08-18

**Authors:** Ana Carolina Ottaviani, Diana Quirino Monteiro, Camila Rafael Ferreira Campos, Elizabeth Joan Barham, Déborah Oliveira, Keila Cristianne Trindade da Cruz, Larissa Corrêa, Fabiana de Souza Orlandi, Marisa Silvana Zazzetta, Aline Cristina Martins Gratão, Sofia Cristina Iost Pavarini

**Affiliations:** ^1^Department of Gerontology, Universidade Federal de São Carlos, São Carlos, São Paulo, Brazil; ^2^Postgraduate Programme in Psychology, Universidade Federal de São Carlos, São Carlos, São Paulo, Brazil; ^3^Department of Psychology, Universidade Federal de São Carlos, São Carlos, São Paulo, Brazil; ^4^Department of Psychiatry, School of Medicine, Universidade Federal de São Paulo, São Paulo, São Paulo, Brazil; ^5^Department of Nursing, Faculty of Health Sciences, Universidade de Brasília, Brasília, Brazil; ^6^Postgraduate Programme in Nursing, Universidade Federal de São Carlos, São Carlos, São Paulo, Brazil

**Keywords:** caregivers, dementia, internet-based intervention, user-centered design, usability, acceptability

## Abstract

**Objective:**

To assess usability and acceptability of *iSupport-Brasil* (*iSupport-BR*) to virtually support family caregivers of people who have dementia.

**Materials and methods:**

In the first stage, nine caregivers/former caregivers assessed the interface of the platform that hosts *iSupport-BR*. In the second stage, 10 caregivers assessed acceptability of the platform and answered the System Usability Scale (SUS), which varies from 0 to 100 points. A descriptive analysis of the quantitative data was performed, as well as a thematic analysis on the open questions. All the ethical aspects were respected.

**Results:**

The results of the first stage indicated a user-friendly interface of the system and relevant content of the program, with 55.6 and 77.8% of the participants assigning the maximum grade to these questions, respectively. Of the five possible points, the system’s mean score was 3.7. In Stage 2, 80% of the caregivers rated the program as very useful and 100% would recommend it to other caregivers. Perception of the program’s usability by the SUS scale was excellent (*M* = 86.5 ± 11.5).

**Conclusion:**

This research allowed elaborating the final version of *iSupport-BR*, considering usability and acceptability of the platform and the program for computers/notebooks, being a pioneer in evaluating it for use in smartphones. Future research studies will have to assess the effects of *iSupport-BR* on the caregivers’ mental health.

## Introduction

More than 55 million people have dementia in the world, and a number of projections indicate that there will be nearly 78 million by 2030 and 139 million in 2050 (1). Most people with dementia are cared for by family members or other unpaid caregivers, especially in countries with limited resources where there is shortage or lack of formal support services for dementia (1).

Family caregivers of people living with dementia, most of whom are women, are frequently exposed to multiple stressors that affect physical and mental health, including high task demands, physical and emotional exhaustion, financial problems and limitations in social and leisure activities, in addition to dealing with the effects of dementia on the family member’s well-being (2, 3). Offering the practical and effective support means to this population is essential to prevent and mitigate such impacts (4).

Online programs can be a support strategy with positive effects for caregivers (5, 6), and may even be a viable alternative to traditional face-to-face support modalities, as it has the potential to reach a larger target audience by overcoming geographic barriers, and for being cheaper, flexible, and adaptable to the caregivers’ routines (4, 7, 8).

Systematic reviews show that online interventions for family caregivers of people who have dementia can be effective in increasing psychological well-being, helping to reduce depression, anxiety and stress symptoms resulting from caregiving, through improved self-efficacy, satisfaction, confidence and care skills (6, 7, 9–11). The social distancing resulting from the COVID-19 pandemic has corroborated the use of new technologies to support families in need of care, especially those who cannot participate in face-to-face activities (12, 13).

However, interventions offered in virtual format and using an Internet connection may present some challenges related to technology (design, usability, level of digital literacy and acceptability), as well as organizational (lack of information about the intervention, gap between research and implementation), socioeconomic (lack of funding and cost of technology) and ethical (privacy, mechanization of care) (14). Satisfactory usability and acceptability levels are related to better involvement and coping in the use of new technologies (15, 16).

A review study on the usability and acceptability of Internet-based interventions developed with caregivers of people who have dementia shows that, for the most part, they are effective, efficient and satisfactory, as well as being considered useful and acceptable by this population (17). Involvement of the target audience in the process of developing and implementing new technologies that use the Internet is extremely important to improve usability and acceptability of the program (14). A number of research studies indicate that caregivers of people who have dementia, for example, prefer to receive online support through audio and video, as it is the strategy that best suits day-to-day care and generates greater satisfaction and well-being (16, 18). Considering such preferences is fundamental so that efficacy of the program for this population segment can be improved.

Development and implementation of supportive interventions that are accessible, acceptable and effective for informal caregivers of people who have dementia is a strategic priority in the Global Action Plan for the Public Health Response to Dementia 2017–2025 (19). In this perspective, the World Health Organization (WHO) has developed iSupport for Dementia, an online program that aims at providing education, skills training and support for family caregivers of people who have dementia (20). The iSupport program presents 23 lessons distributed across five modules, which cover well-established topics on caregivers’ care and self-care.

The initial version of iSupport was developed in English using an encompassing and multicultural approach for transcultural adaptation in several countries, such as Portugal (21), Australia (22), India (23), Netherlands (24), and Switzerland (25). In Brazil, the iSupport cross-cultural adaptation process was carried out as part of a multicenter study funded by the National Health Fund, in partnership with the Ministry of Health, following all WHO recommendations.

iSupport has the potential to improve universal access to education and support for family members so that they can better deal with socio-emotional issues that arise from interacting with a person who has dementia (26). In the context of online interventions, programs with high usability and acceptability can reduce barriers to use, improve user experience and engagement, and prevent program abandonment (27). In this way, evaluating usability and acceptability, as well as exploring the users’ perceptions about the iSupport version adapted for the Brazilian context - *iSupport-Brasil* or *iSupport-BR* - makes it possible to understand the feasibility of a global and evidence-based resource for use with/by caregivers of people who have dementia in Brazil. The objective of this study was to evaluate usability and acceptability of *iSupport-BR*, exploring the perceptions of caregivers of people who have dementia about benefits, facilitators and barriers to using the program in a preliminary study.

## Materials and methods

A mixed-methods study was carried out, involving a usability test stage of the system interface (Stage 1) and a subsequent preliminary usability and acceptability evaluation of *iSupport-Brasil* by caregivers (Stage 2). The procedures adopted were based on the adaptation guide made available by WHO to the licensees. The guide offers standardized advice for translating and culturally adapting iSupport to fit the local target group while guaranteeing that the adapted version is equivalent to the generic one. The usability and acceptability tests allow noticing the users’ interaction with the system (28), with direct implications for the later dissemination and use of the program. They can include techniques for the collection of qualitative and quantitative data. Due to the COVID-19 pandemic, the study was entirely conducted online.

### Characteristics of the *iSupport-Brasil* platform

The content included in the iSupport program culturally adapted to the Brazilian context was incorporated to a digital platform, as described below.

**FIGURE 1:**
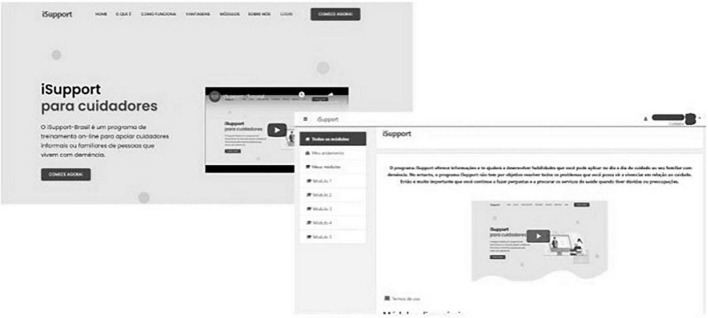
Mock-up landing page of the platform (screen to the left) and page to search *iSupport-Brasil* (*iSupport-BR*) modules (screen to the right).

The process of introducing content into an online platform and evaluating the interface, usability and acceptability of the Portuguese version of the iSupport program was the result of a partnership between Brazilian researchers from the Federal University of São Carlos (*Universidade Federal de São Carlos*, UFSCar), the University of Brasília (*Universidade de Brasília*, UnB) and the Federal University of São Paulo (*Universidade Federal de São Paulo*, UNIFESP). An Information and Communication Technology company was hired to develop the digital platform. All maintenance tasks and code-related problems were in charge of the company. After testing several options, the *Moodle* platform was adopted because it is free, with a greater community of developers and users, facilitating its maintenance and continuous and unrestricted use. The platform is in the domain of the UFSCar. The platform’s registration is linked to the Federal Ministry of Health to ensure greater visibility and universal free access by people^1^. iSupport was originally created to be self-applied. From the home page, registered users can access the program at any time and from any place. A Welcome page, which the users visit before login, presents an overview of the program and asks the person to enroll (Figure 1).

Together with the team of developers, the researchers defined that the platform should provide the user with an account with a login and password, access to information in the form of modules and to the activities of each module of the manual for validation of learning. The platform also stores the users’ answers in the activities in the form of a database. Therefore, the system requirements that have been established are user login (caregivers); administrators login (for managing the platform content); CRUD (Create, Read, Update, and Delete) of users of both profiles; CRUD of teaching modules (text, video and audio); visualization of teaching modules; Testing CRUD for teaching validation; and storage of the users’ answers.

The platform was filled-in with the content included in iSupport, aiming at the best layout of the content in the system, so that it presents good usability and is accessible (29). Each user can customize their support plan according to their needs, choosing which of the five modules to access, being able to follow progress of the modules through the percentage bar of the content covered so far. The activities include diverse information regarding text, illustrative images, care settings, and interactive skill exercises. Immediate feedback is provided on the answers to the exercises.

The *iSupport-Brasil* platform is flexible (the user chooses to visit the activities they consider most relevant), personalized (the name of the person who cares, the care recipient, and the degree of kinship are used in the text of the platform to make the material as close as possible to the user), interactive and responsive (the user resorts to their knowledge and experience to answer the questions and to apply what they have learned to their personal situation, and the notes taken are recorded; feedback is given according to the answers marked), scalable (it is possible to insert or remove content instantly and it can be used by an unlimited number of people), secure (after completing registration, it is necessary to go through a simple registration validation process); and responsive to the user’s technology (it adapts to different screen sizes – computer, tablet, or smartphone).

### Stage 1 – Usability test of the system’s interface

#### Recruitment and sample

The interface usability test took place from 11/23/2020 to 12/04/2020 and the objective was to assess the caregivers’ perception on the *iSupport-Brasil* platform’s interface. Caregivers and/or former caregivers were recruited based on the following eligibility criteria: (a) being 18 years of age or older; (b) identifying themselves as family caregiver or former caregiver of a person who has (or had) dementia in the last 6 months (self-reported diagnosis); and (c) having access to a computer, smartphone, or tablet, with an Internet connection. Recruitment was in charge of the company itself *via WhatsApp* and email, means through which the individuals were informed about the research objective and stages. From a contact list of the researchers and the company, 15 family caregivers/former caregivers of people who have dementia were invited to evaluate the platform before it was made available to test its usability and acceptability. Six of them refused to participate in the study, totaling a convenience sample comprised by nine participants. After acceptance, to assess functionality of the platform and whether people would be able to use it effectively, a usability test questionnaire was created using the Typeform™ software. The test created by means of Typeform™ allows access through a computer or any mobile device.

#### Methods and variables

At a first moment, the participants filled out a form with sociodemographic information (gender, age, schooling, and marital status) and care context (type of dementia, age of the person who has/had dementia). Questions about the use and previous experiences with electronic equipment were also asked, including the following: devices commonly used to access the Internet (computer, smartphone and tablet) and apps used on such equipment (e.g., *WhatsApp*, *YouTube*, search in *Google*, email, *Facebook*, *Instagram*, *Netflix*, *Spotify*, and *Google* Agenda).

**FIGURE 2:**
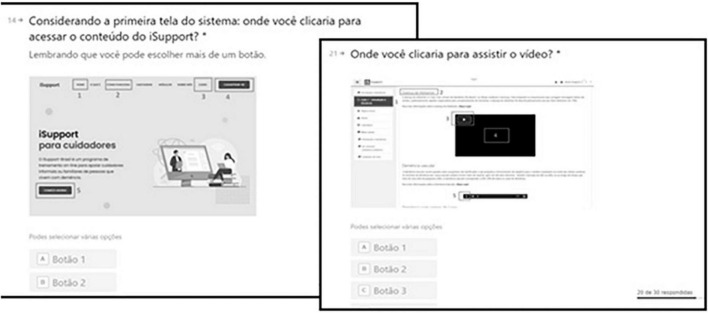
Mock-up of the assessment of access to the content (screen to the left) and how to watch a video (screen to the right) in the *iSupport-Brasil* (*iSupport-BR*) version.

Evaluation of the performance in the interface tasks was carried out through the presentation of clippings from the *iSupport-BR* homepage in which the participants indicated how they performed the tasks, including: how they would (a) access the content of *iSupport-Brasil* (Figure 2, left), (b) register a new user, (c) start the first module, (d) start the first activity, (e) identify the name of the first activity, (f) go to the next activity, (g) identify different content formats (text, audio, video, and link), (h) watch a video (Figure 2, right), (i) start an audio, (j) answer a form, and (k) send the answer and track progress of the activity.

In addition to that, a subjective evaluation was carried out for the participants to indicate their first impressions regarding the appearance of the program using a Likert scale from 1 to 5 to evaluate: (a) simplicity of the system (1 = very difficult to use; 5 = very easy to use); (b) appearance of the system (1 = I didn’t like it; 5 = I liked it a lot); and (c) usefulness (1 = not very useful; 5 = very useful). In the last question, the participants answered which of the five *iSupport-Brasil* modules interested them the most. The company contacted the participants throughout the data collection period to help them access the platform and the questionnaire, as well as to obtain immediate feedback on the system.

### Stage 2 – Preliminary usability and acceptability assessment of *iSupport-Brasil*

#### Recruitment and sample

The researchers recruited caregivers based on the following eligibility criteria: (a) being 18 years of age or older; (b) identifying themselves as the current family caregiver of a person who has been living with dementia for at least 6 months (self-reported diagnosis); and (c) having access to a computer, smartphone, or tablet with Internet access. From a contact list of the researchers, 22 family caregivers of people who have dementia were invited to use and evaluate the platform, of which seven refused to participate and three were no longer caregivers. The participants were recruited *via WhatsApp*, when they were informed about the study objective and stages. After accepting to receive more information about the study, the Free and Informed Consent Form was made available through the *Google Forms* platform for reading, agreement and acceptance. The study participants were 12 caregivers, although only 10 composed the convenience sample and completed all the stages.

#### Methods and variables

• A form with diverse information on sociodemographic (gender, age, schooling, and marital status) and care context (type of dementia), age of the person who has dementia aspects. Questions about use and previous experience with electronic equipment, including the following: devices used to access the Internet (computer, smartphone, and tablet), use of electronic equipment (use of mouse, adjust volume/brightness/screen size, email or *WhatsApp*, accessing links [websites], starting and stopping videos) and level of difficulty using electronic equipment (scale from 0 to 10, with 0 being very easy and 10 being very difficult).
• Individual perception of the system: a questionnaire prepared by the research team, containing diverse information on usability: level of satisfaction with the program (from 0 = very dissatisfied to 10 = very satisfied) and questions in relation to the perceptions about the system regarding its complexity, ease, parts of the program that they liked the most/least, navigation, learning, confidence to use (1 = strongly disagree to 5 = strongly agree). The following were considered to assess acceptability: perception of usefulness (from 0 = not very useful to 10 = very useful), recommending the program to other caregivers (1 = yes, 2 = no), reasons that hindered using the program (lack of time/privacy/interest, death or change of residence of the person who has dementia, unexpected demands, difficulty concentrating, and no reason), preference regarding the program’s format (online, with professional, and printed), other suggestions and how they organized to use the program (open answer). This questionnaire was developed based on the review study by Ottaviani et al. (17), in which it was investigated which variables are relevant for usability and acceptability in Internet-based interventions for caregivers of older adults.
• System Usability Scale (SUS): developed by Brooke (30) and translated into Brazilian Portuguese by Teixeira (31), consisting of 10 items evaluated on Likert scales with 1–5 points (from 1 = strongly disagree to 5 = I fully agree). In the odd questions, one point has to be subtracted from the score referring to the user’s answer. In the even questions, five points have to be subtracted from the answer. Subsequently, all values of the ten questions must be added and multiplied by 2.5. The final score varies from 0 to 100, with higher values representing better usability. A cutoff point of 68 was used in this research to define a mean usability score above or below the cutoff point (30, 31).

#### Data collection procedure

After signing the Free and Informed Consent Form, the sociodemographic and care form, as well as questions about previous experiences with electronic equipment, were sent to the participants. Subsequently, the link to access and register in the *iSupport-BR* program was provided, which the users should employ for a 3-week period. After this period, the caregivers were contacted again to fill in one last electronic form with questions on the individual perception of the system and the SUS scale (both *via Google Forms*).

#### Data analysis

The data were exported to *Excel*^®^ and the quantitative data were imported into the *Statistical Package for Social Sciences* (SPSS) statistical software, version 21, for data analysis and presentation. We adopted a qualitative descriptive design and used open-ended questions to collect participants’ general experiences and attitudes toward the usability and acceptability of the program. For open questions, thematic content analysis was performed to identify key themes. The analysis was performed by two researchers, ACO and DQM, who, after becoming familiar with the data by reading and re-reading the responses, started to identify the core topics. The themes found were discussed with the entire team in meetings, until reaching a consensus (32).

### Ethical aspects

All the ethical procedures for research with human beings were respected. Ethical approval was granted by the Research Ethics Committee of the UFSCar, as a multicenter study, under the following opinions: 2,647,432, 3,154,538, 3,251,479, and 3,628,919. Before initiating data collection, all the participants read and agreed to the Free and Informed Consent Form (FICF) and to the Secrecy Form, online and *via Google Forms*.

## Results

### Characteristics of the samples

A descriptive analysis was performed to evaluate the quantitative data. In stage 1, all participants (*n* = 9) were women (*n* = 100%), most were married/in stable relationships (*n* = 44.4%), with predominance of the age group from 50 to 59 years old (*n* = 55.6%) and with nine to 12 years or more of schooling (*n* = 88.8%); among the people with dementia, most were between 80 and 89 years old (*n* = 66.7%). In stage 2, the sample (*n* = 10) presented similar characteristics, with predominance of women (*n* = 90%), married/in stable unions (*n* = 70%), age group from 50 to 59 years old (*n* = 50%), with 12 years or more of schooling (*n* = 80%) and who assisted people living with dementia in the age group from 80 to 89 years old (*n* = 70%).

### Stage 1 – Usability test of the system’s interface

**FIGURE 3:**
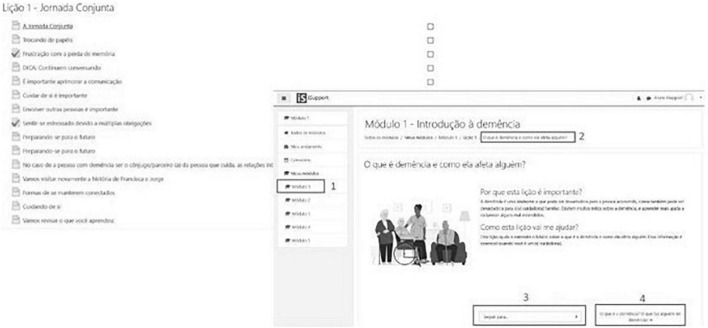
Mock-up of the screen to start the first activity (to the left) and the screen with the end of the first lesson (to the right) of the *iSupport-Brasil* (*iSupport-BR*) version.

The assessment showed that the system has a user-friendly interface (55.6% assigned score 5) and relevant content (77.8%, score 5). Of the five possible points, the mean overall score assigned to the system was 3.7. Common difficulties with the interface included the following: finding the “next” button in the activities (Figure 3, to the right), with the name “*Lição*” (“Lesson” 9; Figure3, to the left), and the small font size of the texts. To correct the suggestions made by the participants, a green background was placed on the “next” and font increase buttons throughout the entire platform. Nearly 55.6% (*n* = 5) considered “Module 5 - Dealing with changing behaviors in a person with dementia”, as the most interesting of all modules, followed by module 4 - Providing everyday care (44.5%; *n* = 4) and Module 3 – Caring for me (33.3%; *n* = 3).

### General perceptions of the users about the platform’s interface

**FIGURE 4:**
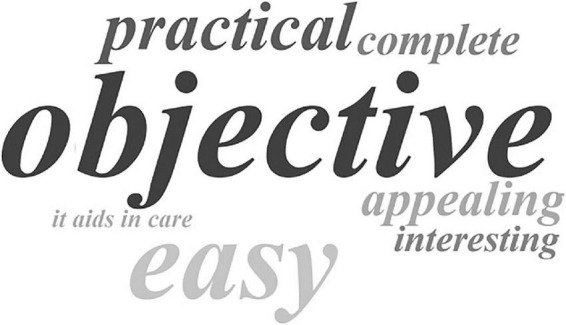
Word cloud showing the adjectives used to describe the impressions about *iSupport-Brasil* (*iSupport-BR*).

Through informal verbal feedback, all caregivers indicated that the program was pleasant to use, that the content was easy to understand, and that it met the expectations and assisted in the care provided. Figure 4, prepared through an online word cloud art-making platform^2^, shows the adjectives most used by the participants to describe the program, which included appealing, complete, easy, interesting, objective and practical.

### Stage 2 – preliminary usability and acceptability assessment of *iSupport-Brasil*

The total score of the SUS scale was 86.5 (±11.5), suggesting excellent usability. Tables 1, 2 present the data about the usability and acceptability assessment.

**TABLE 1:** Usability assessment of the *iSupport-Brasil* program, Stage 2 (*n* = 10), 2021.

Variable	Category	*n* (%)
Satisfaction level	Very satisfied	6 (60.0)
	Moderately satisfied	4 (40.0)
	Not at all satisfied	–
The program’s structure is complex (difficulty navigating)	I totally disagree	5 (50.0)
	I disagree	5 (50.0)
*The program is easy to use	I totally agree	5 (50.0)
	I agree	5 (50.0)
*I would need help from someone with technical knowledge to use the *iSupport-BR* program	I totally disagree	5 (50.0)
	I disagree	4 (40.0)
	I agree	1 (10.0)
*The components of the *iSupport-BR* program are very well integrated	I totally agree	4 (40.0)
	I agree	5 (50.0)
	Neutral	1 (10.0)
*Navigation in the *iSupport-BR* program presents many inconsistencies (the way of using the program varied from one module to another)	I totally disagree	5 (50.0)
	I disagree	5 (50.0)
*Most people would quickly learn how to use the *iSupport-BR* program	I totally agree	3 (30.0)
	I agree	5 (50.0)
	Neutral	2 (20.0)
*I would use *iSupport-BR* frequently (until finishing the lessons that I am interested in)	I totally agree	6 (60.0)
	I agree	4 (40.0)
*I feel confident enough to use the *iSupport-BR* program	I totally agree	7 (70.0)
	I agree	3 (30.0)
*I need to learn many new things before I can use the *iSupport-BR* program	I totally disagree	6 (60.0)
	I disagree	4 (40.0)

*Only the categories with answers are indicated in the table.

**TABLE 2:** Acceptability assessment of the *iSupport-Brasil* program, Stage 2 (*n* = 10), 2021.

Variable	Category	*n* (%)
Usefulness level	Very useful	8 (80.0)
	Moderately useful	2 (20.0)
	Not at all useful	–
I would recommend *iSupport-BR*	Yes	10 (100.0)
	No	–
Barriers that hinder use	Lack of time	3 (30.0)
	Unexpected situations/requirements	2 (20.0)
	Difficulty concentrating/Tiredness	1 (10.0)
	No reason	4 (40.0)
Format of the program	Online	8 (80.0)
	With a professional	1 (10.0)
	Printed (Book)	1 (10.0)

Most of the caregivers indicated being very satisfied (60.0%) and considered the program as very useful (80.0%). In general, the caregivers indicated that the program was easy to use and navigate, in addition to being well-integrated, and that most people would be able to learn how to use it. The majority felt confident enough to use the program, with no need to develop new skills, and they would use it frequently (until finishing the lessons they are interested in). All caregivers also answered that they would recommend the *iSupport-BR* program to other caregivers and most of them indicated a preference for doing the program in the online format – which is the current format. The following stood out among the barriers that hindered use: 30.0% mentioned lack of time; 20.0% care-related unexpected situations; and 10.0%, difficulty concentrating.

The suggestions on how to improve usability refer to the following: (a) visual inconsistencies (title written twice, click on audio when it is video); (b) lack of feedback on a lesson; (c) lack of kinship customization code in some lessons; (d) unidentified/unknown icons (e.g., icons for texts and activities), (e) non-clickable elements that appear clickable (e.g., track your progress); (f) lack of back/forward button in the pages; and (g) percentage of some activities that did not show 100% when completed. All these items were improved.

### The caregivers’ perceptions about the program through open answers

In general, the participants highlight positive aspects of the content included in *iSupport-BR*, such as:

“*I’ve just finished Module 1; in this first module I found that the content was really objective with extra links for more information. It was good!”* (Caregiver 3).

*“I really liked it, I learned a lot as a caregiver myself”* (Caregiver 4).

*“The content is really complete and easy to understand”* (Caregiver 8).

*“I found it really interesting”* (Caregiver 9).

When asked how they organized themselves to use *iSupport-BR*, considering time, space and other conditions, most of them indicated doing the lessons in the care intervals, at night before going to bed. They also emphasized limited time availability, as well as a desire to being able to do more activities.

*“I always did the lessons in the computer, in my room, at the breaks”* (Caregiver 1).

*“The program is perfect. I adapted and reconciled my time and schedules without any problem”* (Caregiver 2).

*“I found it practical to access through the cell phone, I use it more at night when things are calmer”* (Caregiver 3).

*“I did most of the lessons at night, before going to bed”* (Caregiver 4).

*“Little time needed and I used my cell phone to access it”* (Caregiver 6).

Finally, some caregivers made comments to improve usability of *iSupport-BR*, such as:

“*I think that the bottoms to answer and send the answers to the questionnaires are very repetitive. In this sense, usability could be more intuitive. In addition to this, a more intuitive organization of my notes, PDF files, the meditation audios and lesson summaries could help bring back specific passages for everyday tasks”* (Caregiver 1).

*“I’d only like that there was a change in the color when you finish a lesson or module”* (Caregiver 2).

*“In the future, having the option of having a handout”* (Caregiver 10).

## Discussion

Both qualitatively and quantitatively, this study evaluated usability and acceptability of the translated and adapted version of *iSupport-Brasil*, exploring the perceptions, benefits, facilitators and barriers for using the program in the preliminary study. In general, the program was considered acceptable and usable by the family caregivers, presenting excellent usability and satisfaction levels. Regarding the interface, the participants stated that it was easy to use, that the content was easy to understand, and that it fulfilled their expectations, in addition to assisting in the care provided. The usability and acceptability test results showed good outcomes in task performance, satisfaction and program usefulness, with an excellent perception of program usability.

Our findings are consistent with the study carried out by the iSupport research team from Portugal, in which usability of the program showed success rates above 80% in the tasks. A mean score of 89.5 in the SUS scale, from a cutoff value of 68 points (33), also showed an excellent usability perception in the Portuguese version. The program was considered reliable and the participants were satisfied with its aspect and ease of use (27). Despite the positive feedback regarding *iSupport-Brasil*, we noticed the need to implement improvements in the program in terms of style and aesthetics (visual inconsistencies, icons and non-clickable elements), as well as functional requirements (lack of feedback, of the customization code of the kinship degree, and of the percentage of activities completed in some tasks). In the *iSupport-Portugal* version, some improvement suggestions were also indicated, such as the program’s presentation, functional requirements, format of the content and topics of the classes (27).

Although there is a need for improvements, the literature shows that having good performance in the tasks, together with positive evaluations by the participants about the ease of use of products, services and interactive electronic environments (eSystems), suggests that most usability problems are not serious, although they must be solved for a more satisfactory user experience and efficient navigation (34). For this reason, global dementia policies recognize the potential benefits of digital health in achieving universal health coverage, but emphasize the importance of measuring its usefulness and affordability before its widespread implementation (26).

The limited support of a small size of participants from the family and target context can be extended only in part to other limited supports, with the presence of limited resources from the iSupport group that will be quickly contributed to our data. In addition, group compositions vary within other groups, which may contribute to reducing biases, such as the effect of social desirability. Most of the participants in both stages of the study had high schooling (≥12 years) and previous experience in using electronic equipment with the Internet; therefore, the results do not reflect the perceptions of caregivers with lower schooling levels and who probably have less digital literacy (35, 36). In fact, Teles et al. (21) point out that it is possible that iSupport is more frequently used by a specific segment of caregivers of people who have dementia, such as more educated caregivers, secondary caregivers (less involved with care than primary caregivers) and caregivers with concomitant formal work activity. In order to ensure acceptance and dissemination of technology use, mainly among the oldest and low-schooled caregivers, Tsai et al. (37) suggest that digital health support should therefore be integrated to the interventions that involve Internet use.

Our research focused on a restricted and specific cultural and health environment in Brazil, although a diverse group of participants was included in terms of age and care context, only one male participated and one caregiver had four or fewer years of schooling. Schooling is a determining factor for Internet use. The lack of incentives in countries where Internet use by caregivers of people who have dementia is potentially lower; for example, in low- and middle-income countries, it represents an important gap, as almost 70% of the people who have dementia currently live in such countries and their family caregivers are mostly unsupported (19). However, we decided to use qualitative methods to extract and explore in depth the nature of barriers in the acceptance and use of iSupport at the local level. Similar approaches are needed in all configurations and contexts where iSupport is being deployed.

This study has some limitations that should be considered. Our research focused on a restricted and specific cultural and health environment in Brazil, although a diverse group of participants was included in terms of age and care context, only one male participated and one caregiver had four or fewer years of schooling. Schooling is a determining factor for Internet use. The lack of incentives in countries where Internet use by caregivers of people who have dementia is potentially lower; for example, in low- and middle-income countries, it represents an important gap, as almost 70% of the people who have dementia currently live in such countries and their family caregivers are mostly unsupported (19). In addition, our findings are limited to a small sample size and may be extended to other contexts only in part. The group compositions vary within other groups, which may contribute to reducing biases, such as the effect of social desirability. The use of qualitative methods was important to extract and extract and explore in depth the nature of barriers in the acceptance and use of iSupport at the local level. Similar approaches are needed in all configurations and contexts where iSupport is being deployed.

It is hoped that this study will help to enhance the positive effects of *iSupport-BR*, as it made it possible to evaluate and improve acceptability and usability of the Brazilian version of this program. It is also hoped that this study can contribute to the well-being of countless people who will benefit from this initiative, meeting the global goals of supporting individuals affected by dementia. This initiative may also support the formulation of technological strategies to help solve other social and health challenges and the implementation of a care policy for caregivers of people who have dementia in Brazil. A future study of the efficacy and effectiveness of *iSupport-Brasil* will be able to verify to what extent an online platform is able to help this population, promoting their quality of life and mental health.

## Conclusion

The Brazilian version of iSupport was considered acceptable and usable by family caregivers of people who have dementia in the preliminary study, presenting excellent usability and satisfaction levels, and positive subjective perceptions about the program. The caregivers’ impressions reinforce the importance of the participation of possible users in the development of new electronic support programs. Brazil is the first country in Latin America to implement the cross-cultural adaptation of the iSupport program, and was even a pioneer in the use of the program on smartphones, and it can therefore be an example for other countries that are adapting this program or other online interventions.

## Data availability statement

The raw data supporting the conclusions of this article will be made available by the authors, without undue reservation.

## Ethics statement

The studies involving human participants were reviewed and approved by Research Ethics Committee of the Universidade Federal de São Carlos. The patients/participants provided their written informed consent to participate in this study.

## Author contributions

AO, DM, CF, EB, DO, KC, LC, FS, MZ, AG, and SP conceived, designed, drafted, and revised the manuscript. All authors have read and agreed to the published version of the manuscript.

## Footnotes

1
isupport.saude.gov.br

2
www.wordart.com
